# A High Prevalence of Autism Spectrum Disorder in Preschool Children in an Immigrant, Multiethnic Population in Sweden: Challenges for Health Care

**DOI:** 10.1007/s10803-020-04563-8

**Published:** 2020-06-12

**Authors:** Petra Linnsand, Christopher Gillberg, Åsa Nilses, Bibbi Hagberg, Gudrun Nygren

**Affiliations:** 1 Gillberg Neuropsychiatry Centre, Sahlgrenska Academy, University of Gothenburg, Kungsgatan 12, 411 19 Gothenburg, Sweden; 2 Child and Adolescent Specialist Centre, Angered Hospital, Halmtorget 1, 424 65 Angered, Sweden; 3 Center for Progress in Children’s Mental Health, Kungsgatan 12, 411 19 Gothenburg, Sweden

**Keywords:** Autism, Prevalence, Immigrant population, Health care

## Abstract

This study examines the prevalence of autism spectrum disorder (ASD) in preschool children in an immigrant population. Possible risk factors for ASD and individual needs for the children and their families are described, as well as implications for health care. The estimated minimum prevalence for ASD in the area was 3.66% for children aged 2–5 years. Multiple risk factors and extensive individual needs for the children and their families were observed. The high prevalence of ASD and the plethora of needs in immigrant communities pose challenges for health care. A coordinated health care system is necessary to meet the many and individual needs.

## Introduction

Autism Spectrum Disorder (ASD) is a neurodevelopmental disorder characterized by social communication impairments and behavioral restrictions. These deficits originate in early childhood and impact everyday functioning (American Psychiatric Association 2013). The importance of identifying ASD at an early age has been highlighted in many studies (Coleman and Gillberg 2012; Fernell et al. 2013; Magan-Maganto et al. 2017; Zwaigenbaum et al. 2015). A comprehensive assessment, preferably by an interdisciplinary team, is essential (Gillberg 2010), and a reliable diagnosis can be made by 24 months of age in many cases (Sanchack and Thomas 2016). Co-existing conditions are very common (Carlsson et al. 2013; Gillberg 2010; Gillberg and Billstedt 2000; Posserud et al. 2018; Reinvall et al. 2016; Soke et al. 2018). In 2010, Gillberg (2010) introduced the concept of ESSENCE (Early Symptomatic Syndromes Eliciting Neurodevelopmental Clinical Examinations) to provide an umbrella term for the overlapping and coexisting neurodevelopmental disorders or developmental problems, including ASD in early childhood.

The number of reported cases of ASD has increased over the past 30 years and the current prevalence has been estimated to be at least 1.5% in developed countries (Lyall et al. 2017).

Studies from different countries have suggested the prevalence of ASD, especially in combination with intellectual disability (ID), to be higher among children of immigrant than non-immigrant women (Abdullahi et al. 2018, 2019a; Bolton et al. 2014; Fairthorne et al. 2017; Gillberg et al. 1987; Keen et al. 2010; Magnusson et al. 2012; Wing 1993). In a systematic review, a higher prevalence of ASD was shown in children who had migrant mothers, 2.69% (mothers mostly from African, Asian or Caribbean countries), compared to children of non-migrant mothers, 0.9% (Crafa and Warfa 2015). Kawa et al. (2017) have also found a higher prevalence rate among children of immigrants compared to native born children in 15 of 17 studies conducted in Europe (range of RR 1.02–1.74; OR 0.6–10.5). In contrast, evidence of lower prevalence was reported for children born to immigrant Hispanic women in the U.S, suggesting delayed services for these children (Baio et al. 2018).

The etiology of ASD is multifactorial (Fernell et al. 2013; Ng et al. 2017), and genetic factors play a major role (Lai et al. 2014). The genetic and other factors, as well as gene-environment interplay may contribute to ASD phenotypes (Coleman and Gillberg 2012). The non-genetic factors most commonly studied in association with ASD are sociodemographic characteristics (e.g. advanced parental ages and birth order), physiological factors (clustering of pregnancy and birth complications, e.g. pre-eclampsia, diabetes, caesarean section, trauma, intrauterine hypoxia or birth asphyxia, neonatal jaundice, preterm birth, and low birth weight), and chemical factors, such as traffic-related air pollutants (Coleman and Gillberg 1985; Gillberg 1980; Gillberg and Coleman 1992; Modabbernia et al. 2017; Ng et al. 2017; Wang et al. 2017). The mother’s status as foreign-born is regarded as a risk factor (Gillberg et al. 1987; Guinchat et al. 2012; Lotter 1978; Modabbernia et al. 2017; Ng et al. 2017; Wing 1993). The understanding of the role of environmental factors in ASD etiology is still largely in its infancy, and future research should continue exploring how different factors may be interrelated (Ng et al. 2017).

The reason for the association between maternal migration and the risk of having a child with ASD is not clear. The uterine environment might be affected by stress-induced epigenetic changes during pregnancy and delivery. Epigenetic risk factors associated with ASD include poor pregnancy conditions, low birth weight and congenital malformations (Crafa and Warfa 2015). There are indications that early exposure to inadequate levels of vitamin D and cholesterol may interact with other factors and contribute to the etiology of ASD (Fernell et al. 2015; Gillberg et al. 2017; Magnusson et al. 2016; Mazahery et al. 2016).

The results regarding the association between socioeconomic status (SES) and ASD are conflicting (Delobel-Ayoub et al. 2015; Rai et al. 2012; Thomas et al. 2011; Windham et al. 2011). The conflicting results are likely in part related to available health care. However, the results are more consistent when intellectual level is considered. Delobel-Ayoub et al. (2015) have found a higher prevalence of ASD with ID in areas with lower SES. Also, the findings for ID and childhood disabilities in general have showed an increased prevalence among children from lower socioeconomic backgrounds (Durkin et al. 2017; Spencer et al. 2015).

In Sweden, a large proportion of the population has foreign background, defined as being born abroad or born in Sweden with both parents born abroad. Around 2543 400 (24.9%) inhabitants of Sweden were of a foreign background in 2018 (Statistikmyndigheten 2020). According to the Swedish Medical Birth Register, 25.5–28.4% of the babies born between 2013 and 2016 had mothers who were born abroad. Of the mothers, 14.9–17.6% were born in Asia and Africa (Socialstyrelsen 2020).

Gothenburg is a city in the western part of Sweden, and the second largest city in the country, with a population of ~ 564,000 inhabitants in 2018. In Gothenburg, ~ 198,000 (35.1%) of the inhabitants have foreign background. The largest foreign-born populations residing came from Iraq (2.2%), Iran (2.2%), Somalia (1.5%), and Bosnia and Herzegovina (1.3%) (Statistik och Analys Göteborgs stad 2018).

In an earlier study from Gothenburg, the prevalence of ASD in a general population of 2-year-old children has been reported 0.8% (Nygren et al. 2012a). According to current available statistics from health care in Gothenburg, the prevalence of ASD among children 2–5 years was estimated 1.03% (unpublished data 2018).

The aim of this study was to examine the prevalence of ASD in preschool children, in one of the most multiethnic immigrant population districts of Gothenburg. The complexity as regards possible risk factors and the individual needs for children and their families will be described, and implications for health care discussed.

## Methods

### Study Area

The study area in this study consists of one district of Gothenburg, with ~ 13,200 inhabitants of whom ~ 1350 were children aged 0–5 years at the end of 2018. The district is one of the most multiethnic and socioeconomically disadvantaged areas in Gothenburg. In the study area, 89.6% of the population has foreign background. The most common birth countries are Iraq (10.3%), Somalia (5.5%), Syria (5.4%), and Bosnia-Hercegovina (5.1%). The area has high prevalence rates of ill health, high levels of unemployment and low average income (Statistik och Analys Göteborgs stad 2018). Sweden has a very high Human Development Index (HDI) (United Nations Development Programme 2018), but in the study area ~ 56% of the foreign-born population has a medium/low/unknown HDI (Statistik och Analys Göteborgs stad 2018).

### Child Health Center

Sweden has complementary health care for all children. The local Child Health Center (CHC) in the study area provides a developmental surveillance program for children between 0–6 years and reaches about 99% of all children (Central Barnhälsovård Västra Götalandsregionen 2018). The public health services in the city of Gothenburg have implemented a screening programme, to enable early detection of ASD. All 30-month-old children are screened for ASD at the CHC with a combination of instruments: the parental questionnaire Modified Checklist for Autism in Toddlers—Revised with Follow-up (M-CHAT-R/F) and the clinical observation of the child, the Joint Attention-Observation (JA-OBS) (Nygren et al. 2012b). The methods are also used if there is suspicion of ASD prior to routine screening.

### Target Population

The target population for this study consisted of all children born during the period of January 1, 2013 to December 31, 2016, and registered at the CHC in the study area on December 31, 2018 (n = 902, 454 males, 448 females).

### Procedure for the Comprehensive Neuropsychiatric Assessment

Since 2013, there is a local collaborative assessment and intervention program with a multidisciplinary team for families with young children with neurodevelopmental problems in the study area. This enables both assessment and interventions close to the family’s local setting, in collaboration with the health care nurse at the CHC, the preschool staff and other social services. The team is located in a family center, which includes the CHC, an open preschool,^1^ the maternity health care and parental support provided by the social welfare. The goals for the local program are to provide assessment and individually planned interventions to children with neurodevelopmental disorders in their own neighborhood, to increase the accessibility and continuity in the health care provided, as well as parents’ involvement in the interventions.

Children identified at risk for ASD or other neurodevelopmental disorders at the CHC are referred to the local multidisciplinary team for further assessment and interventions. The multidisciplinary team consists of a pediatrician/child psychiatrist, a developmental psychologist, a special education teacher, a speech and language pathologist, a child specialist nurse, and a social worker. A professional interpreter was needed in about 60% of the assessments. The multidisciplinary assessment for all children included: (1) A physical developmental examination, hearing test, blood tests, including SNP array, and other relevant investigations according to the pediatrician’s assessment; (2) A parental interview regarding the child’s developmental and medical history, current clinical symptoms and social situation; (3) The ESSENCE-Q-REV (Gillberg 2010); (4) The Wechsler Preschool and Primary Scale of Intelligence, Fourth Edition (WPPSI-IV) (Wechsler 2012) for the children over 2:6 years, and for younger children Merrill-Palmer—Revised Scales of Development (MP-R) (Roid and Sampers 2004); (5) The Autism Diagnostic Observation Schedule Second Edition (ADOS-2) (Lord et al. 2012); (6) The Vineland Adaptive Behavior Scales Second Edition (VABS-II) (Sparrow et al. 2005); (7) An observation of the child in his or her preschool.

The ASD diagnosis was based on all obtained information from the assessments, and distinguished in accordance with the criteria of the DSM-5 (299.00, Autism Spectrum Disorder) (American Psychiatric Association 2013).

### Measures

#### Prevalence Estimates

The prevalence of ASD was estimated for children born in 2013–2016 and registered at the CHC in the study area, on December 31, 2018.

The first author (PL) and the nurses at CHC reviewed all the medical/health care records of registered children born during this period, and all children with a diagnosis of ASD were included.

#### Data Collection from Medical Records

Medical records from the CHC and the comprehensive neuropsychiatric assessment were scrutinized by two of the authors (PL and GN) independently. Parameters recorded included demographics, maternal country of birth, timing of migration to Sweden, prenatal and perinatal history, heredity, genetic analyses, and results of the diagnostic assessment.

#### Risk Factors

Four systematic reviews were used to identify potential risk factors for developing ASD (Crafa and Warfa 2015; Modabbernia et al. 2017; Ng et al. 2017; Wang et al. 2017). The identified risk factors have been grouped according to the following classification: geographical region of maternal birth, other prenatal risk factors, perinatal risk factors, neurodevelopmental disorders in the family, and genetic findings/syndromes, see Table 1.

**Table 1:** Classification into five domains of potential risk factors for ASD

Geographical region of maternal birth	Maternal country of birth, studied by geographical region of birth Categorized according to United Nations definitions (available at https://unstats.un.org/unsd/methodology/m49): Eastern Africa, Northern Africa, South/Eastern Asia, Western Asia, and Northern Europe
Other prenatal risk factors^a^	a. Advanced parental age (parental aged ≥ 40) b. Parity (first or ≥ fourth) c. Clustering of pregnancy complications, including maternal health problems, such as gestational diabetes, preeclampsia, abnormal thyroid function, infections, and mental health problem; and maternal prenatal medication, such as antibiotics
Perinatal risk factors^b^	a. Clustering of birth complications, such as intrauterine hypoxia or birth asphyxia, including 5 min Apgar scores < 7; elective and emergency caesarean section; and assisted birth, including vacuum extraction and forceps b. Preterm birth, gestational age ≤ 36 weeks c. Small size for gestational age (SGA), > 2 SD below the mean birth weight for the gestational age according to Swedish birth weight standards d. Other medical conditions during the neonatal period, such as infections and neonatal jaundice
Neurodevelopmental disorders in the family	A positive family history was defined as a child having a first or second degree relative (full siblings, parents, grandparents, aunts, uncles) affected by: a. ASD b. Other neurodevelopmental disorders, such as language disorder, ADHD, and epilepsy
Genetic findings/syndromes	a. In SNP array potentials causative copy number variations b. Medical syndrome suspected/Minor physical anomalies (MPA) c. Consanguinity

^a^Prenatal period defined as the period of gestation

^b^Perinatal period defined as the period immediately before and after the birth and to 4 weeks after birth

Regarding prenatal and perinatal risk factors, the comparison data were obtained from the Swedish Medical Birth Register. The register contains prospectively collected data from antenatal and obstetric care, as well as demographic information, on 99% of all Swedish births since 1973 (Socialstyrelsen 2020). The children in the study were compared with children born in 2016 and registered in the Region Västra Götaland (n = 19 937). Pregnancy outcomes, including preeclampsia, caesarean section, Apgar score at 5 min, gestational age, and growth for gestational age, were retrieved.

#### Maternal Migration

In addition to maternal country of birth, timing of migration in relation to the child’s birth was recorded. Three categories were used: (a) the migration occurred ≥ 1 year before the child’s birth; (b) in the year before birth; (c) after birth. Migration status was also studied by level of human development (using the UNDP Human Development Index, HDI) (United Nations Development Programme 2019).

#### Cognitive Level of Children Diagnosed with ASD

The cognitive level of the children was categorized as follows: average intellectual functioning = IQ/Developmental Quotient (DQ) ≥ 85 (AIF), borderline intellectual functioning = IQ/DQ 70–84 (BIF), and intellectual disability (ID) in cases with IQ/DQ < 70, and corresponding adaptive criteria. On the basis of formally performed testing, the results of the cognitive level (IQ/DQ) were found in 21 children (67.7%). For the remaining children, an estimation of developmental level was made, based on the psychologist’s observation during the test situation, results of the VABS-II (Sparrow et al. 2005), as well as other clinical observations during the different parts of the child’s assessment.

### Statistical Analyses

The results are presented descriptively, counting the number of children, and by presenting means, standard deviations, and proportions.

## Results

### Prevalence Estimates

#### Children Diagnosed with ASD

On December 31, 2018, 33 children (24 males, 9 females), born between 2013 and 2016, were identified at the CHC with a diagnosis of ASD according to DSM-5 (American Psychiatric Association 2013). In the target population of 902 children, the male: female ratio was about 1:1 (454 males, 448 females). The male: female ratio for children with ASD in this population was 2.6:1. Of these 33 children, 31 received their diagnosis after assessment in the local team and two children received the diagnosis at another clinic.

The children diagnosed with ASD by the local team included 31 children, 23 boys (74.2%) and eight girls (25.8%). All parents that were approached regarding participation in the study gave their informed consent to participate. Average age for the ASD diagnosis was 36.3 months (range = 22–61, SD 8.8). The majority of these children had been referred after the autism screening at the 30-month health checkup. Six children had been referred at an earlier age on the basis of early clinical suspicion of ASD.

All children had co-existing symptoms, other neurodevelopmental disorders and/or other medical conditions, such as sleeping- and eating problems, early ADHD symptoms, and/or epilepsy. Two children (6.5%) were shown to have AIF, 9 children (29%) had BIF, and 20 children (64.5%) had ID.

### Prevalence Ratios

The prevalence of ASD was estimated at 3.66% (33/902 children) for children aged 2–5 years on the census day. For the males, the prevalence of ASD was estimated at 5.29% and for the females 2.01%. The prevalence rates and number of children diagnosed with ASD in different age groups are displayed in Table 2.

**Table 2:** The prevalence of ASD in different age groups in the study population (n = 902)

Year of birth	Number of children registered at CHC	Number of children diagnosed with ASD (m:f)	Prevalence of ASD (m:f) (%)
2013	227	12 (8:4)^a^	5.29 (7.01:3.54)
2014	229	13 (11:2)	5.68 (9.82:1.71)
2015	234	4 (2:2)^a^	1.71 (1.71:1.65)
2016	212	4 (3:1)^a^	1.89 (2.65:1.01)

^a^Number of children on census day. After this day, six children with ASD were identified and diagnosed. Including these children, the prevalence for ASD in 2–5 year old children in the immigrant population would be 4.32%

#### Risk Factors for ASD

According to the used classification, potential risk factors were identified for all children within at least two domains. In 15 children (48.4%), risk factors were identified in three areas, and in seven children (22.6%) within four areas. The most common risk factors were maternal migration status (90.3%) and other prenatal risk factors (77.4%). In the study group, 58.1% of the children had a first and/or second degree relative affected by ASD and/or other neurodevelopmental problems. In Table 3, potential risk factors for each child are displayed.

**Table 3:** Potential risk factors for ASD

Geographical region of maternal birth	Other prenatal risk factors (a–c)	Perinatal risk factors (d–g)	Neurodevelopmental disorders in the family (h–i)	Genetic findings/syndromes (j–l)
Eastern Africa	b			
Eastern Africa	b		h	
Eastern Africa			i	Data missing
Eastern Africa	b	d, g	i	
Eastern Africa	b	f		
Eastern Africa		d		
Eastern Africa	b		i	
Eastern Africa	b, c	d, e, f, g	i	
Eastern Africa		g	h	
Eastern Africa	c		i	Data missing
Eastern Africa	b	d	h, i	
Eastern Africa	a	f	i	
Northern Africa	c	e, f		k
Northern Africa	b, c	d		
South/Eastern Asia	b, c	d	i	
South/Eastern Asia	b	d, e, g	i	
South/Eastern Asia	b	d		
South/Eastern Asia	a, b			
South/Eastern Asia	c		h	
South/Eastern Asia	b	d		
South/Eastern Asia	b			k
Western Asia			i	l
Western Asia	b		i	
Western Asia		e		
Western Asia		d	i	
Western Asia				k, l
Western Asia	b			
Western Asia	a			
Northern Europe	b		h, i	j, l
Northern Europe	b, c	g	i	
Northern Europe	b		i	

a Advanced parental age (parental aged ≥ 40)

b Parity (first or ≥ fourth)

c Clustering of pregnancy complications, including maternal health problems, such as gestational diabetes, preeclampsia, abnormal thyroid function, infections, and mental health problem; and maternal prenatal medication, such as antibiotics

d Clustering of birth complications, such as intrauterine hypoxia or birth asphyxia, including 5 min Apgar scores < 7; elective and emergency caesarean section; and assisted birth, including vacuum extraction and forceps

e Preterm birth, gestational age ≤ 36 weeks

f Small size for gestational age (SGA), > 2 SD below the mean birth weight for the gestational age according to Swedish birth weight standards

g Other medical conditions during the neonatal period, such as infections and neonatal jaundice

h A first or second degree relative affected by ASD

i A first or second degree relative affected by other neurodevelopmental disorders

j In SNP array potentials causative copy number variations

k Medical syndrome suspected/Minor physical anomalies (MPA)

l Consanguinity

#### Geographical Region of Maternal Birth

Of the mothers, 12 (38.7%) were born in Eastern Africa, two (6.5%) in Northern Africa, seven (22.6%) in South/Eastern Asia, seven (22.6%) in Western Asia, and three mothers (9.7%) were born in Northern Europe. Twenty-five mothers (80.6%) had migrated more than a year before the child’s birth, and three mothers (9.7%) had migrated the year prior to the child’s birth. Four children (12.9%) were born abroad (Eastern Africa, Northern Africa, South/Eastern Asia, South/Eastern Asia), and one of them was born during the migration process. In relation to the Human Development Index Level, 14 mothers (45.2%) came from regions with a low HDI.

#### Other Prenatal Risk Factors

Beside risk factors in relation to maternal migration, at least one other prenatal risk factor was identified in 24 children (77.4%), and the most common was parity first or fourth or more (61.3%).

The maternal and paternal average age at delivery was 28 years (range = 17–39, SD 5.37), respectively 34 years (range = 21–47, SD 6.13). Three of the fathers (9.7%) were older than 40 years (43–47). Six children (19.4%) were an only child, 9 (29%) were first born, and four (12.9%) were fourth born or later in the order of siblings. The average number of children in the family was 2.5 (median = 2, range 1–6).

For seven mothers (22.6%), a clustering of pregnancy complications was described in medical records. Two mothers (6.5%) were affected by preeclampsia (3.2% of the mothers throughout the Region Västra Götaland) (Socialstyrelsen 2020). Three mothers (9.7%) suffered mental health problems during pregnancy, and one of them reported extremely stressful events during the migration. Other conditions described were hypothyreosis (one mother), and medication with antibiotics due to infection (one mother). All pregnancies were singleton.

#### Perinatal Risk Factors

At least one perinatal risk factor was found in 16 children (51.6%), and the most common was a clustering of birth complications (32.3%).

Twenty-four of the children had been delivered vaginally (77.4%), and in one case the birth had been assisted by vacuum extraction. Seven deliveries were completed by caesarean section (22.6%), of which three were urgent (16.7% of deliveries completed by caesarean section throughout the Region Västra Götaland) (Socialstyrelsen 2020). All children had Apgar scores ≥ 8 after 5 min, or were in good physical health after delivery (according to reports from parents, for children born abroad) (1.1% of the children had Apgar score < 7 after 5 min throughout the Region Västra Götaland) (Socialstyrelsen 2020).

A majority of the children (87.1%) were born after full-term pregnancy (94.4% of the children throughout the Region Västra Götaland) (Socialstyrelsen 2020). Four children (12.9%) were born before 37 weeks gestation (32 + 4 to 36 + 0) (5.6% of the children throughout the Region Västra Götaland) (Socialstyrelsen 2020). Three of the children (9.7%) had a low birth weight, < 2500 g (1700–2200 g) (4.4% of the children throughout the Region Västra Götaland) (Socialstyrelsen 2020), and as many had a birth weight of more than 4000 g (4000–4800 g). Four children (12.9%) were born small size for gestational age (2.5% of the children throughout the Region Västra Götaland) (Socialstyrelsen 2020).

Five children (16.1%) required neonatal care, due to prematurity, feeding difficulties, hyperbilirubinemia, and/or neonatal sepsis.

#### Neurodevelopmental Disorders in the Family

In the study group, five children (16.1%) had a relative diagnosed with ASD. Of these, two were siblings, two children had an older/younger sibling affected by ASD, and one child had an uncle with ASD. Another four children (12.9%) had a sibling with strong suspicion of ASD. Two children (6.5%) in the study group were cousins. Fifteen children (48.4%) had a relative affected by other neurodevelopmental disorders, such as Language disorder and ADHD. Thirteen children (41.9%) had no relatives affected by ASD or other neurodevelopmental disorders, and 22 children (71%) had no relative with ASD or the suspicion of ASD.

#### Genetic Findings/Syndromes

In one child, SNP array analysis showed a deletion (114 kb) on chromosome 10 (uncertain clinical significance). In three children (9.7%), a “medical syndrome” was suspected, due to minor physical anomalies (MPA). In the genetic analyses of three children (9.7%), an increased degree of homozygosity was found, consistent with consanguinity.

## Discussion

### ASD Prevalence

In the current study, the estimated prevalence of ASD among children aged 2–5 years was 3.66%, and this rate is likely to be regarded as a minimum. After December 31, 2018, additional children in the age group had been diagnosed with ASD and the real prevalence is likely to be more than 4.32%. This is higher than the general prevalence reported in many international epidemiological studies (Lyall et al. 2017), as well as in relation to the prevalence of 1.03% among children 2–5 years in the whole city of Gothenburg (unpublished data 2018). However, the results accord well with those of earlier studies from immigrant populations (Abdullahi et al. 2019a; Crafa and Warfa 2015; Fairthorne et al. 2017; Kawa et al. 2017; Magnusson et al. 2012).

The fact that we found a high prevalence of ASD in this sample could be related to several factors. First of all, a universal health care system that reaches all children is important for early identification and diagnosis of ASD. This basic condition was present in the current study, with the developmental surveillance program including autism screening for all children at the age of 30 months at the local CHC. The screening methods, the combination of the M-CHAT-R/F and the JA-OBS, together with the nurses’ level of competence and experience played an important role in identifying the children in need of further assessment. This is in accordance with the findings of Barbaro and Halder (2016) that suggest a combination of instruments, that incorporates parental report and observation components, for early identification of ASD in low resource settings. An earlier population study from Gothenburg has shown a high positive predictive value (90%) for the combination of instruments, and the study confirmed that the screening programme identified ASD in children from immigrant families as well as in children from Swedish families (Nygren et al. 2012b). An indication of a higher prevalence of ASD in children from immigrant families was also reported (Nygren et al. 2012a). We suggest that the combination of methods for early detection of ASD is necessary in areas with a high proportion of immigrant families and in areas with low SES.

Secondly, access to a comprehensive multidisciplinary assessment for diagnosis of ASD is essential (Gillberg 2010). Earlier studies have proposed that immigrant families have more difficulties accessing health care and other services for their children (Carlsson et al. 2016; Lemay et al. 2018; Thomas et al. 2012). In the present study, the local collaborative assessment and intervention program co-located with the CHC, close to the family’s setting, enabled assessment without geographical, organizational or linguistic obstacles.

We argue that the methods for early identification of ASD and access to a comprehensive multidisciplinary assessment are prerequisites for diagnosing ASD in children in the study area. These basic conditions act as confounders in this study, and contribute to the validity of the results.

The reported higher prevalence of ASD in immigrant populations is probably, like ASD itself, multifactorial. Different factors appear to explain the high prevalence of ASD in these populations, such as biological/genetic, non-genetic, as well as epigenetic factors. Thus, higher rate of ASD in immigrant populations are reported across many countries (Abdullahi et al. 2019b; Bolton et al. 2014; Fairthorne et al. 2017; Magnusson et al. 2012), and the risk for ASD with comorbid ID was highest if maternal country of birth was in the African or Asian continents (Abdullahi et al. 2018). In our study, 90% of the children had a mother born in the African or Asian continents, and 64.5% of the children had comorbid ID. A recent study by Abdullahi et al. (2019a) has shown that more severe presentations of ID in children with ASD in immigrant groups allow them to be referred earlier for assessment. This is also likely to reflect the high prevalence of ASD in our group. Crafa and Warfa (2015) have suggested that ethnicity and biological risk factors alone cannot explain the difference rates of ASD found in children born to migrant mothers. Migrations theories of ASD have proposed that environmental factors associated with parents’ migration process contribute to the development of ASD in their offspring, particularly for those with comorbid ID (Magnusson et al. 2012). In our study group, three children (9.7%) were born in the year before the migration, and two of them had ID. Due to limited material, we cannot draw any conclusion from this finding. Migration is often a stressful life event, and there are stressors related to maternal migration that can influence the quality of pre- and perinatal periods. The stressors can have the potential to cause epigenetic changes during the pregnancy and delivery with the associated risk of ASD (Crafa and Warfa 2015).

In a systematic review of epidemiological surveys of ASD, Elsabbagh et al. (2012) have found that, in particular, in low and middle-income countries, there are no data available on the prevalence of ASD. Thus, future studies may demonstrate if the increased risk for ASD in children of immigrant women born outside Europe, Australia or the United States is due to factors affected by the migration itself or may be explained by differences in the prevalence of ASD in different populations.

### Risk Factors of ASD

The exact etiology of ASD remains unclear, and there is lack of consistency in terms of association between specific risk factors and ASD. Genetics has a key role in the etiology, together with environmental factors during the child’s early development. In our study group, an array of risk factors was observed, including maternal immigrant status, clustering of pregnancy and birth complications, heredity, and genetic factors. An increasing number of studies highlight maternal migration as a risk factor for ASD (Crafa and Warfa 2015; Ng et al. 2017), and this was also the most common risk factor in our study group. The link between ASD and migration is still unclear. Several other hypotheses have been proposed concerning the role of migration in ASD, but have not yet been thoroughly tested. These include for example maternal vitamin D deficiency (Mazahery et al. 2016), and maternal stress (Crafa and Warfa 2015). Magnusson et al. (2012) have found that the risk of low-function ASD appeared to be highest when the migration occurred the year prior to birth. This suggests that environmental factors such as a stressful maternal migrations process can affect the neural development and can be considered as a risk factor.

Other known risk factors for ASD are suboptimal pregnancy and birth conditions (Coleman and Gillberg 2012; Guinchat et al. 2012; Ng et al. 2017). In our study group, various prenatal and perinatal risk factors were identified, in addition to maternal migration status, but we cannot evaluate their significance or their potential causal mechanisms, due to the small study group.

ASD has strong genetic influences and twin studies have shown that the heritability is high (Sandin et al. 2014; Tick et al. 2016). In our study group, 29% of the children had a first/second degree relative with ASD or strong suspicion of ASD, and 58.1% had a relative affected by ASD and/or other neurodevelopmental problems. Bolton et al. (2014) have demonstrated that children in an African cohort showed a higher heritability with 36.9% having a positive family history of ASD, compared to 26.3% in the Irish cohort with an ASD diagnosis. Consanguinity has also been considered as a risk factor for ASD, and seems to be more frequent in some cultures (Mamidala et al. 2015). In the present study, an increased degree of homozygosity was found in three children (9.7%).

Discrepancies across studies can partly be explained by heterogeneity of study design and methodological limitations. There has also been variation in the definition of outcomes and variation in the immigration profiles of different countries. This contributes to difficulties in comparing results from different studies in a reliable way. The present study contributes to an in-depth picture of a smaller group of children with ASD in an immigrant population. It shows that there are several risk factors associated with ASD besides the immigrant status of the mother. More epidemiological studies are needed to understand the risk factors in immigrant populations, and possible interactions between these, and smaller studies with more detailed data can be a complement to the epidemiological studies.

### Co-existing Disorders and ID

Coexisting conditions in ASD are very prevalent (Gillberg and Billstedt 2000; Soke et al. 2018), and accumulating studies propose that ASD shares considerable etiology with other neurodevelopmental disorders (Lundstrom et al. 2015). In our study, all children had at least one coexisting condition and 64.5% of the children had ID. In a Swedish study, Kantzer et al. (2013) have reported that 36% of the preschool children with ASD had ID. Bolton et al. (2014) have also found a higher proportion of children with ASD and moderate or severe ID (72.2%) in an African group compared to an Irish group (16.3%). The risk for ASD with ID seems to be higher when families migrated from regions with a low HDI or low-income country (Abdullahi et al. 2019a, b; Esler et al. 2017). In our study, 45.2% of the mothers were born in these regions. This finding raises important questions about potential biological and/or environmental factors that may contribute to the pattern of greater intellectual impairment among children of immigrant parents from regions with low HDI. Several other hypotheses have also been conceptualized to understand the higher frequency of ASD with ID among children of immigrant families, such as vitamin D deficiency, more socioeconomic disadvantages, reduced level of maternal care during the obstetric period in children with an immigrant history, as well as cultural bias in diagnostic procedures related to language barriers and lack of cultural knowledge (Fernell et al. 2015; Haglund and Kallen 2011; Langridge et al. 2013; Magnusson et al. 2012). Abdullahi et al. (2019a) have proposed that children of mothers from foreign-born backgrounds are more likely to be diagnosed younger and to present more severe traits of ASD and associated ID. However, children with ASD and normal IQ may be underdiagnosed in immigrant populations (Fairthorne et al. 2017; Magnusson et al. 2012). In the present target population, there are probably also children with undiagnosed ASD and AIF whose difficulties will become more apparent during the first school years.

### New Models for Children with ASD and Their Families in Immigrant Communities

It is imperative that health care professionals gain knowledge from current research on early symptoms in ASD, treatment approaches and intervention opportunities. At the same time, more knowledge is needed on how cultural values in different populations might influence different views of children’s development in general, as well as ASD.

The families in our study lived in low resource settings. Most of the families had several social stress factors, such as unemployment, separated parents, mental or physical health problems of parents, and in several families, there were siblings with neurodevelopmental disorders. Many families had economic problems and insufficient housing, and some of the families also lacked permanent residency. In addition, all children were affected by co-existing conditions. The co-existing conditions provide an increased need for support from health services and affect the life of the whole family (Petrou et al. 2018; Posserud et al. 2018).

New models are needed in health care to increase accessibility to care, as well as to meet the many and individual needs of young children with ASD and their families especially in immigrant communities. These models need to counteract the gaps in health care. We propose one geographical and organizational unit, with a multi-professional team, for both assessment and interventions for young children with ASD. Treatment approaches to reduce the impact of co-existing conditions whilst providing interventions for ASD are needed, but it is also necessary to take into account the family’s social situation. Vivanti et al. (2018, 2014) have emphasized the importance of understanding and taking into account the child’s characteristics as well as the family’s priorities/expectations, needs, and life situation in the selection of treatment objectives and treatment strategies. The importance of individually tailored interventions pursuant to the child’s needs as well as the parent’s resources and life situation, has been highlighted in a study by Nilses et al. (2019) from our research group.

### Limitations and Strengths

All the children included in the study have been assessed by the same team, consisting of medical, psychological, educational and speech therapy professionals with considerable experience of young children with neurodevelopmental disorders, and consistent diagnostic criteria and diagnostic instruments were used throughout.

Nevertheless, the results of the study must be considered within the context of several study limitations. The prevalence of 3.66% ASD is likely a minimum rate for the area, and the real prevalence is more likely to be higher than 4.32%.

The surveillance area included in this study was a relatively small district of Gothenburg, and the findings only represent this area. With a small number of participants, the results cannot necessarily be generalized to the overall population of immigrant children, and the group is too small for statistical calculations. In addition, research conducted with larger samples and within a wider geographic area is needed.

Data collection methods used in this project relied on the review of records, and results are limited to the information in those records. Certain data were not available, such as maternal vitamin D status, smoking, toxins or illnesses during pregnancy. Therefore, there may be more prenatal and perinatal risk factors than those described in the result.

The strengths of our study include its population-based design. The universal health care system, and the screening programme for ASD in the immigrant area makes it likely that a large proportion of individuals in the community have been identified and diagnosed. There was no dropout in the study. All parents who have been asked have consented to their children’s participation.

Another strength of the study is the focus on an understudied group of children, young children with ASD in an immigrant and low SES community, who are otherwise often excluded from research studies in general.

## Conclusions

A high prevalence of ASD, 3.66%, in children of the immigrant population was found, consistent with findings from some other studies performed over the past 30 years. Multiple risk factors were observed, including maternal immigrant status, clustering of pregnancy and birth complications, family history risk, and genetic factors.

The result highlights the importance of coordinated systems in health care to meet the many and individual needs of young children with ASD and their families. Costless and general care is not enough to reach all immigrant families. Thus, it is of importance that new models be developed so as to increase accessibility to services for children with ASD and other developmental disorders in immigrant communities. Health care professionals need knowledge of neurodevelopmental disorders and how cultural values influence different views of children’s development in general, as well as neurodevelopmental disorders.

Further research must include demographic factors and it is urgent to understand the care-seeking behavior in immigrant populations. Several risk factors exist in the etiology of ASD and need to be further studied.

## Footnotes

1 Open preschool is a meeting place for children and their parents. Preschool teachers are responsible for planning of the activities.
